# Performance Investigation on Different Designs of Superhydrophobic Surface Texture for Composite Insulator

**DOI:** 10.3390/ma12071164

**Published:** 2019-04-10

**Authors:** Meiyun Zhao, Wei Li, Yang Wu, Xinze Zhao, Mingyi Tan, Jingtang Xing

**Affiliations:** 1 Hubei Key Laboratory of Hydroelectric Machinery Design & Maintenance, China Three Gorges University, Yichang 443002, China; sxdxliwei2017@163.com (W.L.); sxdxwuyang2015@163.com (Y.W.); 2 FSI, FEPS, University of Southampton, Southampton SO17 1BJ, UK; M.Tan@soton.ac.uk (M.T.); jtxing@soton.ac.uk (J.X.); 3 National United Engineering Laboratory for Advanced Bearing Tribology, Henan University of Science and Technology, Luoyang 471023, China

**Keywords:** textured surface, superhydrophobic surface, silicon rubber, composite insulator, abrasion resistance

## Abstract

To investigate the superhydrophobic properties of different surface textures, nine designs of textures with micro-nanostructures were produced successfully using the laser engraving technique on the surfaces of composite insulator umbrella skirt samples made of silicon rubber. The optimal parameters of the texture designs to give rise to the best hydrophobicity were determined. The surface morphology, abrasion resistance, corrosion resistance, self-cleaning and antifouling property of the different textured surfaces as well as water droplets rolling on the textured surfaces were studied experimentally using a contact angle meter, scanning electron microscope, three-dimensional topography meter and high-speed camera system. It was found that the diamond column design with optimal parameters has the best superhydrophobicity and overall performance. The most remarkable advantage of the optimal diamond column design is its robustness and long-term superhydrophobicity after repeated de-icing in harsh conditions. The reported work is an important step towards achieving superhydrophobic surface without coating for outdoor composite insulator in practical applications.

## 1. Introduction

Superhydrophobic surfaces have aroused great research interest in recent years because of their application potentials in a variety of engineering fields [1,2,3,4,5]. With the continuous development of material science and bionics, the manufacturing technology of superhydrophobic surface has also become more mature [6,7,8,9]. Currently, two approaches are generally used to fabricate superhydrophobic surfaces. The first is to construct suitable micro-nanocomposite structures with low surface energy directly on the material surface whereas the second is to modify the surface energy of hydrophilic substrate with low surface energy material. No matter which method is adopted, it is necessary to properly regulate the surface microstructure [10].

Silicone rubber is a new polymer material well known for its good properties such as high and low temperature resistance, ageing resistance and good insulation performances, hence it is widely used in many fields, especially in power system insulation devices [11]. Silicone rubber, when used as the composite insulator sheath, needs to have good hydrophobic surfaces since the hydrophobicity plays an important role in anti-icing and pollution flashover resistance [12,13,14].

In the past a few decades a popular strategy to obtain a superhydrophobic surface was to apply a coating with micro-nanostructure on the various substrates and this is widely used in practice because of its high efficiency and easy applications [15,16,17,18]. Many researchers have also developed different superhydrophobic coating materials to be applied on the silicon rubber surface of composite insulators for outdoor uses with noticeable success [19,20,21,22,23]. However, although the coating can improve the surface hydrophobic properties of umbrella skirt of composite insulator and hence can reduce the accumulation of pollutant and droplets, the main concern is whether the superhydrophobic coating can maintain its long-term anti-icing performance in practice. Since coating is an additional material on the substrate surface and the binding strength between the coating and substrate may deteriorate over time, it may separate from the substrate in harsh conditions [24,25,26]. Some researchers have recognised this problem and have developed novel coatings with high binding strength between the coating and substrates [27,28,29], but in harsh conditions or in repeatedly de-icing process the degradation problem of the superhydrophobic surface is still unresolved [30,31,32]. Kulinich et al. [33] have questioned whether the superhydrophobic surface was ice-repellent and have demonstrated through experiments that rough superhydrophobic coatings were not always effective anti-ice surfaces due to their relatively low abrasive resistance and inability to deice in all climatic conditions. Therefore, more attention should be paid to surface microstructure as well as icing mechanisms and conditions. Laser patterning was confirmed to be a promising method due to its high efficiency and suitability for fabricating superhydrophobic surface [34]. Ahmmed et al. [35] fabricated eight different micro-nano superhydrophobic texture samples on the surface of copper in a single-step laser micromachining process to analyze the drag reducing property. This laser texturing method has been widely applied to prepare superhydrophobic metallic surfaces in past years and has gotten good results [36,37,38,39,40,41]. 

With respect to the silicon rubber composite insulator working in outdoor harsh conditions, there are two main disadvantages when superhydrophobic coating is used. The first is that the repeatedly de-icing will destroy the superhydrophobic structure so that the surface will lose superhydrophobicity and it is impractical to change the new insulator frequently [42]. The second is that the coating on the surface of umbrella skirt is easily affected by electric field and other factors in the actual working environment and the coating may also influence the insulating property of the composite insulator. Therefore, in this paper we have adopted the laser engraving technique to engrave different textures on the surfaces of composite insulator, which possesses not only superhydrophobicity, but also good structural integrity. One limitation of our implementation is that the micro structure is not easily engraved on the composite insulator directly, nevertheless, this research can be a significant first step towards achieving textured surface for good performance for the silicon rubber composite insulator. In this paper, nine designs of textures with micro-nanostructures with a range of texture parameters for each design were made using laser engraving technique and the best design with optimal texture parameters was found by comparing the performances of these different textured surfaces. The next step is to optimize the processing method to make superhydrophobic surface on the entire composite insulator.

## 2. Experimental Section

### 2.1. Preparation of the Samples

**Figure 1:**
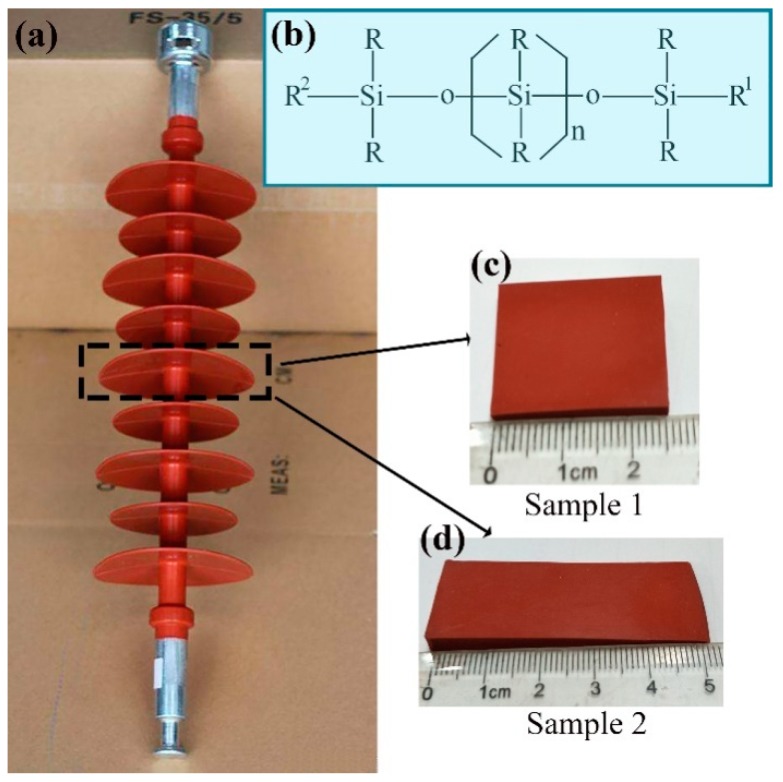
(**a**) Composite insulator; (**b**) silicone rubber molecular formula; (**c**) square sample; (**d**) rectangular sample.

A typical composite insulator is shown in Figure 1a. The umbrella skirt material of the insulator is a type of high temperature vulcanizing (HTV) silicon rubber made of polysiloxane and various additives as well as reinforcing filler through a process of compression, injection moulding and high temperature vulcanization in sequence. The main chain of silicone rubber material is Si–O bond and its chemical structure is shown in Figure 1b. This material is of high chemical and thermal stability, which makes surface laser engraving feasible. The bond energy of Si–O bond can be as high as 451 kJ/mol, far higher than that of C–C (345 kJ/mol) and C–Si (318 kJ/mol), so it requires a higher energy to destroy the bond. The silicone rubber from the umbrella skirt was cut to make 2.5 cm by 2.5 cm square samples and 5 cm by 2 cm rectangular samples as shown in Figure 1c,d. After washing with ethanol and then in water, the samples were dried and stored in plastic bags for subsequent experiments.

### 2.2. Design of Surface Texture

**Figure 2:**
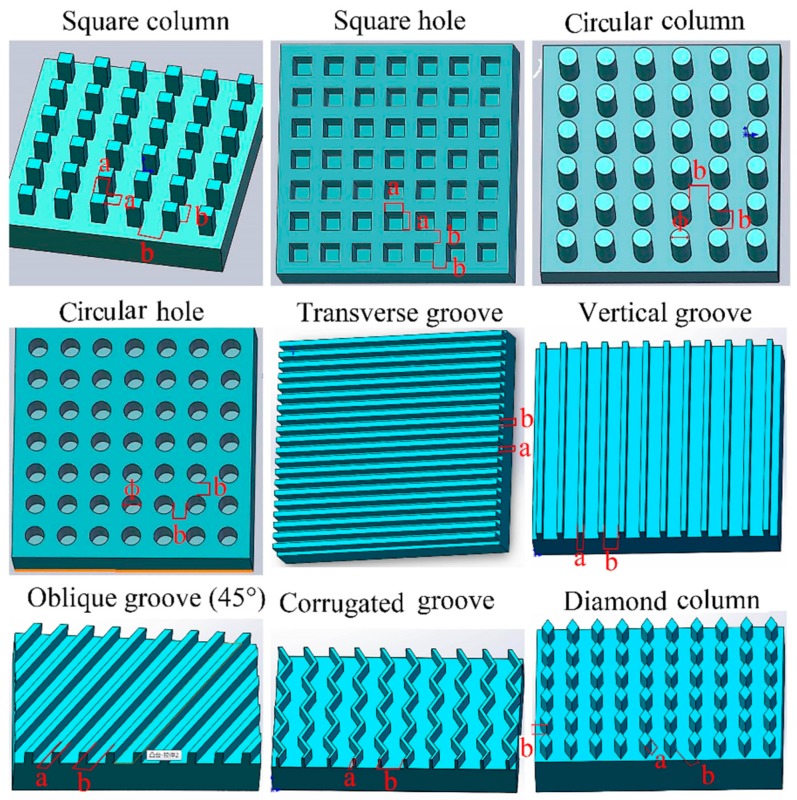
Surface texture designs.

Here D80M multi-function laser engraving machine was used to engrave different textures on the sample surfaces. As shown in Figure 2, nine different texture patterns were designed and they were denoted as square column (SC), square hole (SH), circular column (CC), circular hole (CH), transverse groove (TG) where the direction of the groove was perpendicular to the umbrella skirt angle, vertical groove (VG) where the direction of the groove was parallel with the umbrella skirt angle, oblique groove (OG) where the direction of the groove was at a 45° angle from umbrella skirt, corrugate groove (CG) and diamond column (DC) with an acute angle of 60°. At the same time, in order to study the effect of the size parameters, samples with different texture width represented by the variable *a* in Figure 2, spacing represented by the variable *b* and depth on the surface were made for each texture pattern. Considering the limitation of material properties and the processing method, the values selected for width variable *a* of each texture were 200, 300, 400, 500 and 600 µm and the same values were also adopted for the spacing variable *b*, that is, *b* = 200, 300, 400, 500 and 600 µm. The depth of the texture was controlled by laser engraving power level *P* and velocity *V*. Here 5 power levels, *P* = 15, 25, 35, 45, 55 w, were used, while the engraving speed *V* was fixed at 75 mm/s.

### 2.3. Surface Performance Tests

Composite insulators usually work in a chemically complex environment, such as in acid rain, haze and strong ultraviolet radiation in practical applications. These chemical conditions can erode the surface and possibly cause the surface to lose its superhydrophobicity, so the chemical durability of the textured surfaces should be examined. At the same time, the composite insulator in power transmission system faces all kinds of pollution, and especially in rain and snow weather these pollutants will adhere to the surface of the silicone rubber, which may destroy the surface hydrophobicity and cause pollution flashover. Therefore, the self-cleaning and anti-fouling performance of the composite insulator surface is extremely important. In addition, in the practical working environments, composite insulator may suffer from various mechanical damages, such as de-icing wear, so the wear resistance of the textured surface is an important factor which can affect the mechanical durability of superhydrophobic surfaces [2]. Furthermore, composite insulators often work in cold regions so that they may face the icing problem. The raindrops should fall off the surface as quickly as possible before they are frozen into ice. Therefore, it is important to study the rolling time and speed of the droplets on different texture surfaces. Based on the above, we conducted a series of experiments to verify the performance of the designed textures.

#### 2.3.1. Corrosion Test

An accelerated corrosion test method was adopted to study the effect of acid and alkali corrosive environment on the hydrophobicity of the silicone rubber. The samples were separately immersed in 1 mol/L HCl and 1 mol/L NaOH solution and then taken out every 4 days for testing after being dried in cold wind. Then the contact angle (CA) and the sliding angle (SA) of water droplets on the surface were measured.

#### 2.3.2. Contamination Test

Although the composition of the contamination is slightly different in different regions, its main components are SiO_2_ and NaCl. The SiO_2_ particles in actual dirt are very small, similar to the elementary element of diatomite, so the diatomite was selected as the insoluble matter of SiO_2_ and NaCl was selected as the soluble substances to represent the contamination in the tests. Namely, 0.1 g high purity NaCl (1 mg/mL) and 0.1g diatomite (1 mg/mL) were added to 100 mL slightly acidic HCl solution (pH = 5). A small amount of dextrin (0.01 mg/mL) was also added to the solution during the mixing stage and then the solution was well stirred. In this test, the samples were soaked in the above solution in a container sealed with plastic film to prevent the solvent from volatilization. Every 4 days the samples were taken out, rinsed with water and placed in the laboratory to air dry naturally. Finally, the water CA and the SA of the sample surface were measured.

#### 2.3.3. Wear Test

**Figure 3:**
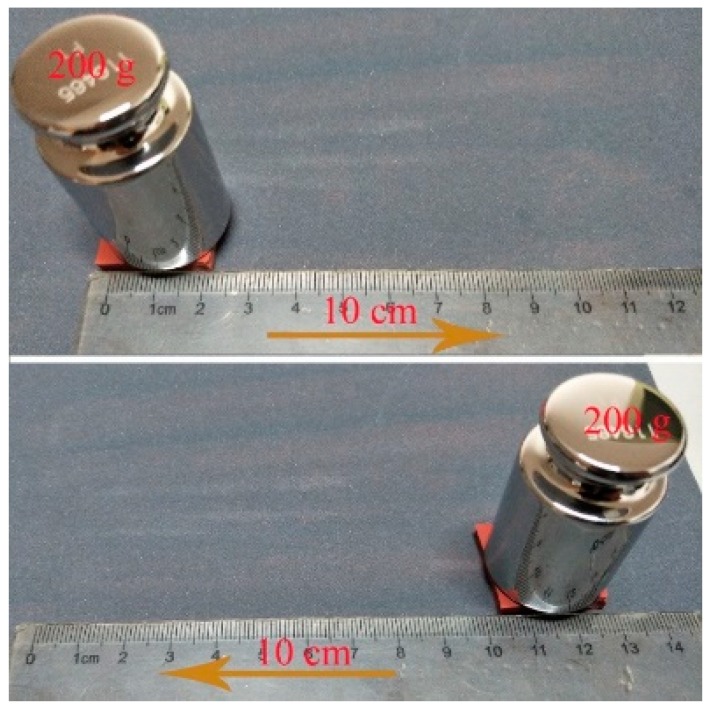
Wear test.

To study the hydrophobicity stability of the textured silicone rubber surface after abrasion, a wear test was conducted. As shown in Figure 3, the textured surface of the samples was placed face-down on a piece of 400 Cw sandpaper and a weight of 200 g was placed on the substrate’s upper surface. An abrasion cycle consisted of a 10 cm forward motion of the substrate along the direction of a ruler and then a backward motion to the original position. After every 5 cycles of abrasion, the textured surface was cleaned and the CA and the SA of the water droplet on the surface were measured. 

#### 2.3.4. Droplets Falling Test

To study the movement of the droplets on different textured surfaces with the different inclination angles of 6°, 12°, 18°, 24° and 30°, the whole process of the droplets’ motion while falling from different heights (3, 5, 6, 9 and 12 cm) was recorded using a high-speed camera system. The results of the bouncing height and the average speed of the droplet motion as well as the state of the droplet movement on the surface were obtained.

## 3. Results and Discussion

### 3.1. Hydrophobicity of Different Textured Surfaces

**Figure 4:**
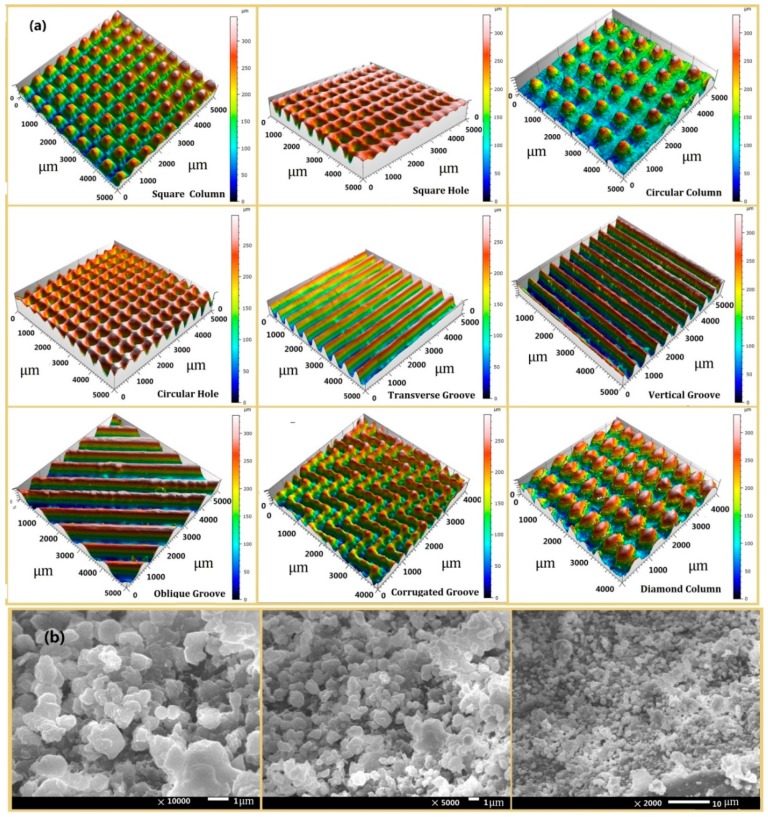
Surface morphology of (**a**) nine textured surface designs and (**b**) SEM images of the square column texture surface.

The CA of the original silicone rubber sample surface without texture is 94.75°, indicating a mere hydrophobic surface. After engraving texture designs on the sample surfaces, surface morphologies were measured by 3D optical profilometer (NANOVEA) and JSM-7500F field-emission scanning electron microscope (JEOL, Toky, Japan) as shown in Figure 4a. The surface hydrophobic properties were measured at room temperature with the JY-PHB contact angle tester (Chengde Jinhe Instrument Manufacturing co., LTD, ChengDe, China) and it was noted that the CAs of all textured surface designs were more than 150°, showing superhydrophobicity. The size of the structures obtained by laser processing is about 200 to 300 microns and these are called primary microstructures since they are greater than 100 microns. Moreover, from the observation of Figure 4b, many nanoscale solid particles exist and some particles gather to form certain group structures on the primary structure with many micron holes, which can be called secondary structures. It is believed that a suitable interplay between the primary and secondary structures is the reason for these textured surfaces to show superhydrophobicity. Data analyses were carried out for all the texture designs with all the parameter combinations, and then the optimal parameters were determined for each design when they showed the best hydrophobicity. The main outcome is summarised in Table 1.

### 3.2. Corrosion Resistance

**Figure 5:**
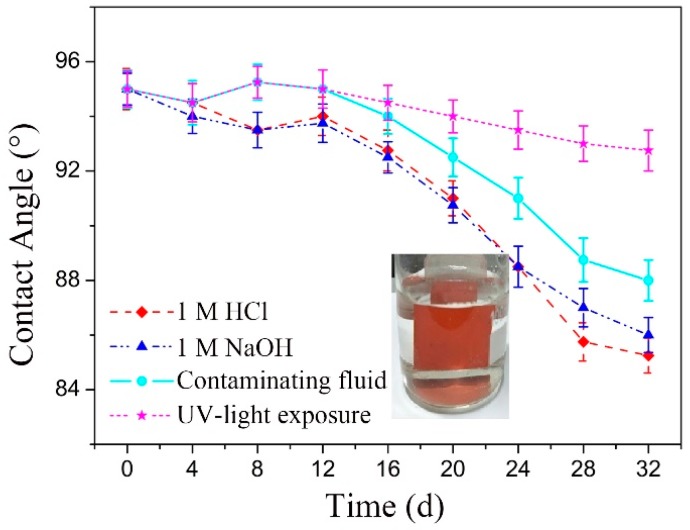
CA curves of the droplet on the original sample over time in different erosion environment.

Figure 5 presents the CA curve variation of the original silicon rubber sample with corrosion time in different corrosive environments. As shown here, the CA values decreased rapidly when the corrosion time was more than 12 days in all the corrosion environments except in UV-light exposure, and the surfaces finally lost hydrophobicity. Therefore, a corrosive environment has a very detrimental effect on the hydrophobicity of a silicon rubber surface although the effect of UV-light is negligible.

**Figure 6:**
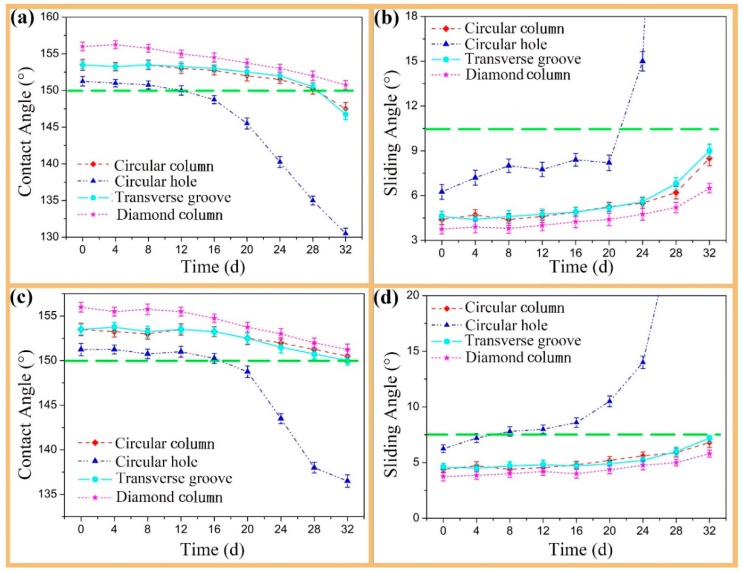
Curves of the CA (**a**,**c**) and the SA (**b**,**d**) of the droplet on different texture designs over time when soaked in (**a**,**b**) 1 mol/L HCl (**c**,**d**) 1 mol/L NaOH.

Figure 6 illustrates the changes of the CA and the SA values of different textured surfaces versus corrosion time in 1 mol/L HCl or 1 mol/L NaOH solution. As shown in Figure 6, the acid has a stronger effect on the textured surfaces than the alkali and both have greater effects on the circular hole design than on other convex structures. For convex textured surfaces, in the whole soaking process of 32 days, the changes of the CA and the SA values are relatively small. It was noted that, with the CA value more than 150° and the SA value less than 6°, the diamond column texture design showed the best superhydrophobicity whether it was in acid or in alkaline solution. So, the diamond column texture design maintained its superhydrophobicity during the whole soaking time. 

### 3.3. Self-Cleaning Antifouling Performance

**Figure 7:**
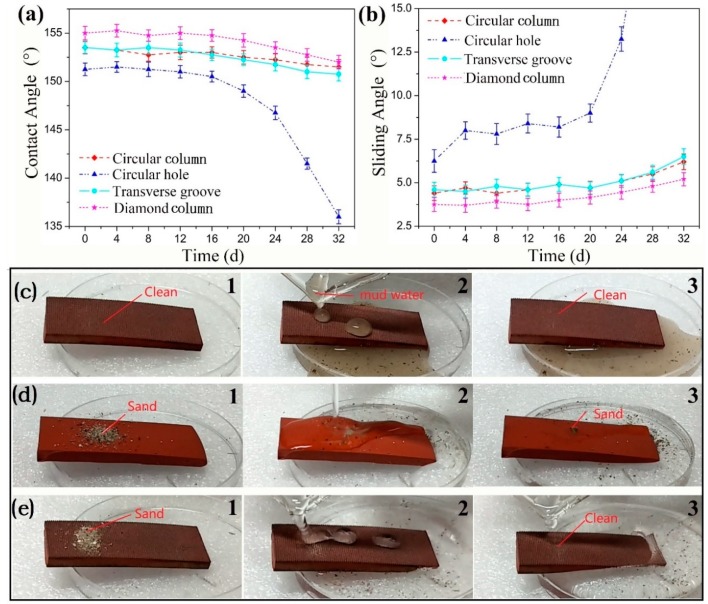
The curves of (**a**) the CAs and (**b**) the SAs of the droplet on different texture surfaces with the time, (**c**) the anti-fouling test of the textured surface with transverse grooves and the self-cleaning test of (**d**) the original sample surface and (**e**) the textured surface with transverse grooves.

Figure 7a,b show the curves of the CA and the SA values of the textured surface designs against the time in the simulated contamination test detailed in Section 2.3.2. The figures illustrate that the CA value of the circular hole textured surface decreased rapidly with the time and the SA increased rapidly, especially when the test time exceeded 20 days. However, for the other convex surfaces, especially for the diamond column textured surface, although the CA declined and the SA increased with the increase of test time, their CA and SA variations were very small and the surfaces still maintained good superhydrophobic performance. This indicates that the convex texture has good antifouling property and can resist the erosion of the contaminants. To further verify the anti-fouling property of the convex textured surfaces, as shown in Figure 7c, muddy water was slowly poured onto the transverse groove textured sample. It was found that the muddy water rolls down from the surface easily without dirt left. In addition, to investigate the self-cleaning performance of textured surface, the fine sediment mixtures were put on the surfaces of the original sample without texture and the textured sample with transverse grooves respectively, then the surfaces were flushed with clean water. From Figure 7d,e, it can be seen that many sand particles remained on the surface of the original sample after being flushed, and some particles were attached to the surface to make it difficult to rinse. However, water easily took away the sand particles on the textured surface with transverse grooves, so the surface became as clean as before, illustrating that the designed textured surfaces have a good self-cleaning property. This self-cleaning property is very beneficial to the working insulators to prevent pollutants from accumulating on the surface of the umbrella skirt, which can reduce pollution flashover and other accidents.

### 3.4. Abrasion Resistance

**Figure 8:**
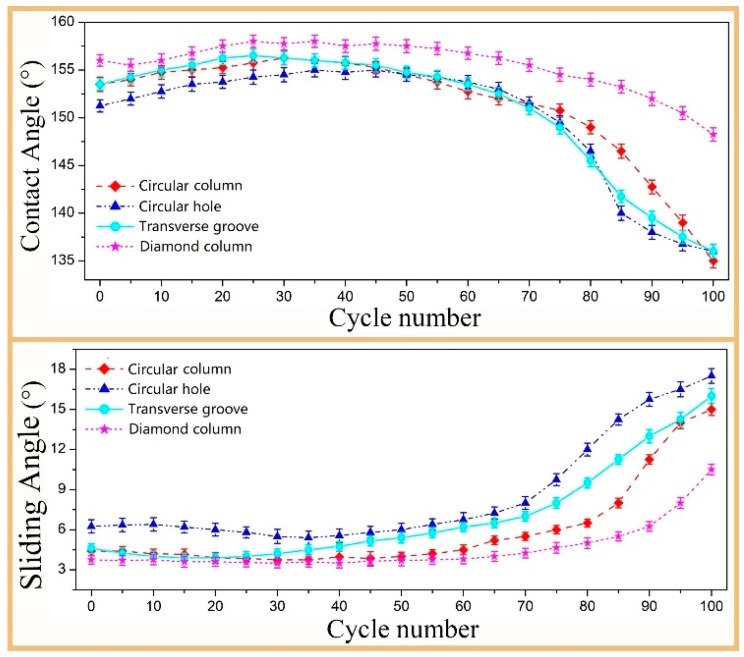
Curves of the CA values and SA values against the abrasion cycles.

**Figure 9:**
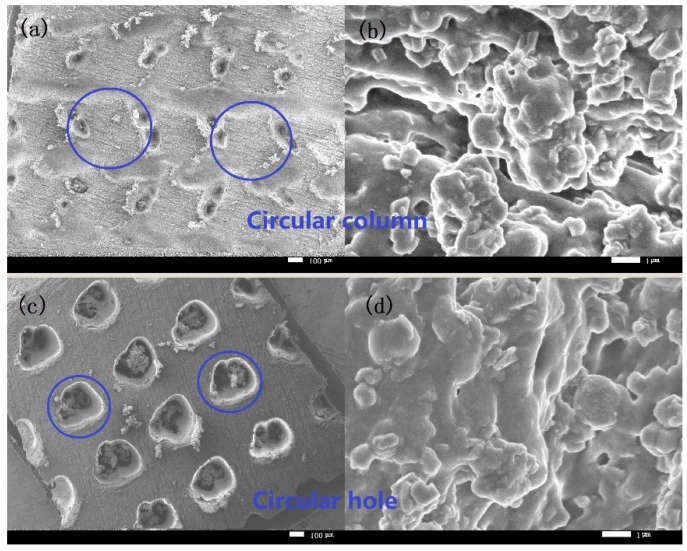
SEM images of (**a**,**b**) circular column and (**c**,**d**) circular hole texture surfaces after 100 abrasion cycles.

Figure 8 shows the curves of the CA and SA values of the different textured surfaces with the number of abrasion cycles. It can be seen from this figure that the CA value of all the textured surfaces increased with the increase of the abrasion cycles in the first 30 cycles, and thereafter the increased abrasion cycles reduced the CA value. When the abrasion cycle was more than 70, the CA values of most textured surfaces, except the diamond column texture surface, declined quickly and finally dropped below 150°; at the same time, the changes in the SA values were very slight with the abrasion cycles up to 70 and then the SA values of most textured surfaces, except the diamond column texture surface, increased rapidly to more than 10°. Figure 9 presents the SEM images of the circular column texture and the circular hole texture surfaces in different magnifications after the 100 abrasion cycles. It was observed that the primary structure of the textures on the surface were destroyed as the number of abrasion cycles increases. The circular column texture becomes smooth and the holes of circular hole texture were filled with abrasion dust, while at the same time, most secondary structures (granular structures) fell off the surface, so the surface became smoother. The results indicate that the surfaces of most texture designs gradually reduce from the initial superhydrophobic state to a hydrophobic state. It is important to note that the diamond column design showed the best wear resistance where the CA values were always greater than 150° and the SA values were less than 8°. In addition, the changes of its CA and SA values were within 5°, even when the abrasion cycles exceeded 100. Only the diamond column design surface kept its superhydrophobicity, whereas the other textured surfaces lost their superhydrophobicity, although they were still hydrophobic throughout the entire abrasion tests. 

### 3.5. Droplet Rolling Performance on Textured Surfaces

**Figure 10:**
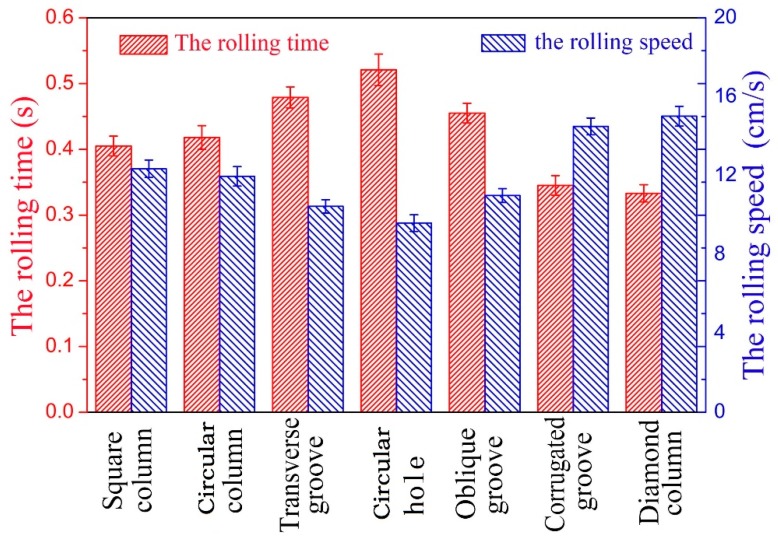
Rolling time and average rolling speed of the droplet dripping from the different texture surfaces.

Table 2 shows the falling time of the droplets on the seven textured surface designs with a declination of 6°, and Figure 10 shows the comparison of the rolling time and the average rolling speed of the droplets acquired by a high-speed camera system. As can be seen from the figure, the rolling time of the droplets on the vertical groove texture surface is the longest and correspondingly its average rolling speed is the lowest. However, the droplet rolling time on the corrugated groove texture and diamond column texture surfaces is only 0.345 ± 0.014 s and 0.333 ± 0.013 s, with the average rolling speed 14.493 ± 0.45 cm/s and 15.015 ± 0.502 cm/s, respectively. The results show that droplets fell off more quickly from the corrugated groove texture and the diamond surface texture under the same conditions. Therefore, these two designs of texture surfaces are more suitable for reducing the icing of composite insulators.

**Figure 11:**
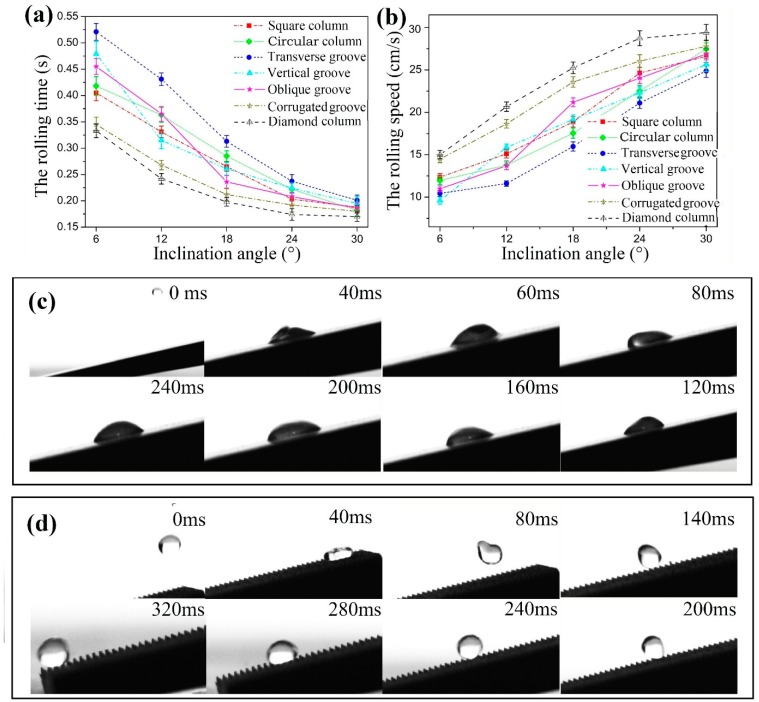
(**a**) Rolling time and (**b**) rolling speed of the droplet dripping on the different textured surfaces against inclination angle and instantaneous snapshots of a droplet dropping onto (**c**) original sample and (**d**) diamond column textured sample.

Figure 11 presents the results of the droplets falling off from different textured surface samples with different inclination angles. As shown in Figure 11a,b, the rolling time of the droplets on all textured surfaces decreased with the increase of the inclination angle, and the decreasing rate gradually reduced. Accordingly, the average rolling speed of the droplets increased with the increase of the inclination angle. It was noted that the rolling time of droplets on the diamond column texture surface was generally the shortest at any inclination angle and correspondingly the average rolling speed was the fastest.

Figure 11c shows the instantaneous snapshots of a droplet dropping from 2 cm of height on the surface of the original untextured sample with an inclination angle of 7.5°, and depicts the process after the droplet was in contact with the untextured sample surface. The droplet almost spread out on the untextured sample surface without rolling down after contact with the surface. Figure 11d shows the instantaneous snapshots of a droplet dropping from the same high on the diamond column textured surface. After 20 ms, the droplet landed on the sample surface and the droplet deformed to be an oblate shape due to the impact force. However, soon afterwards the droplet bounced and became round again to roll down the textured surface. Comparing the two surfaces, it was very clear that the superhydrophobic texture surface is more conducive to the rolling of droplets.

**Figure 12:**
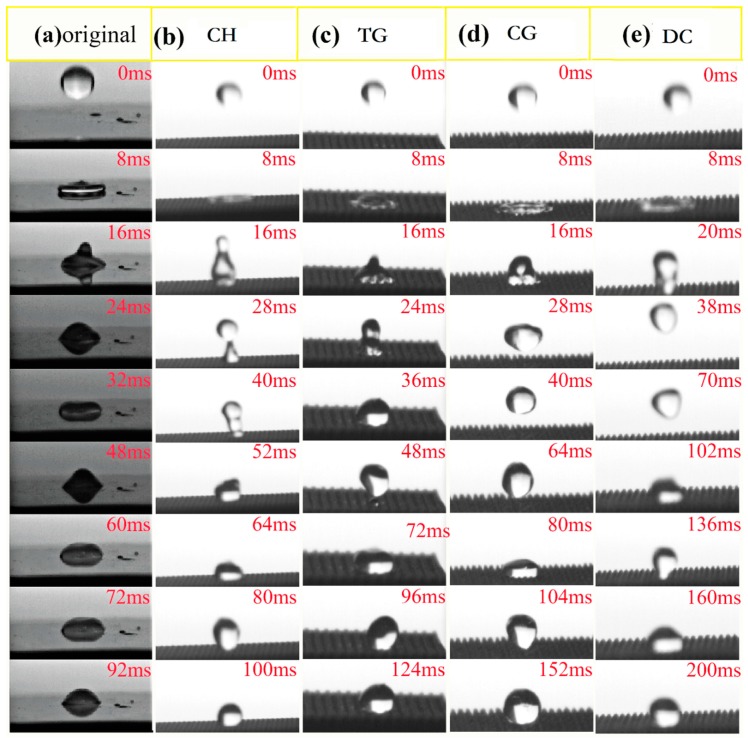
Instantaneous snapshots of the droplet dropping onto the surfaces of (**a**) original, (**b**) circular hole, (**c**) transverse groove, (**d**) corrugated groove, and (**e**) diamond column texture samples.

To compare the behaviours of the droplet bouncing on various textured surfaces, the droplet was dropped freely from a 15 cm height to the sample surface lying horizontally and the whole process was recorded by a high-speed camera. Figure 12 presents the instantaneous snapshots of the droplet dropping onto the original sample surface and the other four textured surface designs previous identified as having longer and shorter rolling times. Figure 12a depicts the process after the droplet fell onto the surface of the original untextured sample surface. As soon as the droplet came into contact with the surface, it almost stuck on the surface and the contact area was quite large. The kinetic energy of the droplet was converted in its own deformation, and the droplet became stationary after just 92 ms. Figure 12b,c show the processes after the droplet fell onto the surfaces of the circular hole and the transverse groove texture samples, and they show similar state changes. The droplets almost spread out when they touch the sample surface in 8 ms. However, the droplets aggregated again at once due to their surface energy and tended to bounce up. Nevertheless, due to the adhesion between the droplet and the surface, it was very difficult for the droplets to bounce wholly off the surfaces and a part of the droplet was firmly attached to the dent structure. Finally, the droplets gradually formed a stable approximate spherical shape on the sample surface after a few milliseconds. Figure 12d,e exhibit the instantaneous snapshots after falling of the droplets onto the surface of the corrugated groove and the diamond column texture samples respectively. It was clear that the situation was very different from those of the previous textured surfaces. Although the droplets did spread on the sample surfaces, they all bounced wholly off the textured surfaces and formed spheres in the air. The droplets then fell onto the surfaces again and became stable spheres after a relatively long-time oscillation. Moreover, the droplet bounced higher on the diamond column textured surface than on the corrugated groove texture surface. The results demonstrate that the adhesion force between the droplet and the diamond column texture surface was the lowest and this texture design is suitable to be applied on the surface of composite insulator to achieve good superhydrophobicity.

## 4. Conclusions

The superhydrophobic surface of the silicon rubber can be obtained by laser engraving and in this work nine designs of textures with micro-nanostructures were successfully made on the surfaces of the composite insulator umbrella skirt samples by laser engraving. The optimal size parameters of all designs were determined based on their surface hydrophobic performance. In order to determine the characteristics of the different textured surfaces including their hydrophobicity, surface morphology, abrasion resistance, corrosion resistance, self-cleaning, antifouling and droplet rolling behaviours on these texture surfaces, a series of experimental studies were conducted using a contact angle meter, scanning electron microscope, three-dimensional topography meter and high-speed camera system. The results obtained in the forms of images, figures and tables clearly illuminate that the diamond column texture surface with optimal sizes has superior superhydrophobicity and overall performance. The optimal surface texture design obtained in this research is an important step towards a solution for outdoor composite insulators achieving long term superhydrophobicity after repeated de-icing in harsh conditions. The outcomes from the experimental investigation reported in this paper are beneficial to both scientific research and engineering applications.

## Figures and Tables

**Table 1:** The optimal parameters of the surface texture designs with the best hydrophobicity.

Texture Designs		SC	SH	CC	CH	TG	VS	OG	CG	DC
Contact Angle (CA)		154°	152°	153.5°	151.25°	153.5°	153°	154°	155°	156°
Sliding Angle (SA)		4.3°	6.5°	4.4°	6.25°	4.6°	5.25°	4.35°	4°	3.75°
Optimal Parameters	*P*/w	35 for all								
	*a*/μm	300	300	400	400	300	300	300	200	300
	*b*/μm	400	200	300	200	300	300	300	300	400

**Table 2:** The rolling time of the droplets on different textured surfaces.

Texture Design	SC	CC	TG	VG	OG	CG	DC
**Rolling Time/s**	0.402	0.418	0.479	0.521	0.455	0.345	0.333
**Measuring Error/s**	0.015	0.018	0.016	0.024	0.015	0.014	0.013
